# Measuring self-control across gender, age, language, and clinical status: A validation study of the Italian version of the Brief Self- Control Scale (BSCS)

**DOI:** 10.1371/journal.pone.0237729

**Published:** 2020-08-21

**Authors:** Francesca Chiesi, Andrea Bonacchi, Chloe Lau, Anna Enrica Tosti, Fabio Marra, Donald H. Saklofske

**Affiliations:** 1 Department of Neuroscience, Psychology, Drug, and Child's Health (NEUROFARBA), University of Florence, Italy; 2 Centro Studi e Ricerca Syntesis, Florence, Italy; 3 Department of Psychology, University of Western Ontario, London, Canada; 4 School of Psychology, University of Florence, Florence, Italy; 5 Experimental and Clinical Medicine Department, University of Florence, Florence, Italy; Universidad de Tarapaca, CHILE

## Abstract

The present study aims to develop and validate an Italian version of the Brief Self-Control Scale (BSCS). A large sample of Italian-speaking participants (*N* = 1139) completed the BSCS and measures of personality and individual dispositions. A clinical sample (*N* = 217) was administered the Italian version and an English-speaking sample (*N* = 274) completed the original version to test measurement invariance. Using confirmatory factor analysis, the best fit was observed for a shortened two-factor model (i.e., impulse control and self-discipline). Metric invariance across languages and partial strong invariance across genders, ages, and clinical status were demonstrated. Internal consistency and test-retest reliability for the total scale were adequate, and validity was established based on its correlations with related constructs and confirming that males and young individuals are more likely to have lower self-control. Results support the use of the shortened BSCS version to assess self-control in Italian-speaking individuals.

## Introduction

Self-control is defined as the capacity to exert conscious control to override impulses and direct responding to promote abstract and distal objectives (e.g., being healthy, obtaining good grades or a professional advancement, to keep out of trouble) when threatened by competing fast, exciting and attractive returns (e.g., to eat unhealthy but tasty food and avoid physical activity, to spend time having fun, to adopt risky behaviors such as alcohol consumption and unprotected sex) [1–3]. As such, differences in this individual disposition (i.e., the capacity to modify dominant responses and to regulate one’s behaviors, thoughts, and emotions) predicts avoidance of inappropriate behaviors that produce strong immediate rewards, and are hence difficult to change or overcome [3–5]. Specifically, lower levels of self-control are associated with a variety of potentially maladaptive behaviors, including unhealthy coping strategies, leading to negative physical and psychological health consequences (e.g., obesity, drug- and alcohol-related problems, sexually transmitted diseases) [3, 5, 6, 7]. Many different theoretical models have been proposed on self-control [5, for a review]; all of which share the assumptions that self-control is conscious and effortful. Moreover, trait self-control is promotes desirable behavior and inhibits undesirable behavior and thus is beneficial for a wide range of life situations.

Hence, a reliable and valid assessment of self-control is deemed of primary importance both for theory (e.g., to better conceptualize the construct) and practice (e.g., for assessing and monitoring the effects of self-control on everyday and clinical outcomes). A number of scales have been developed to assess self-control [for a review, [5, 8]], but rather than assessing self-control across broad behavioral domains in general populations, most scales target specific behaviors in specific populations. Additionally, some measures have become obsolete and have been used infrequently. Thus, de Ridder et al. [5] concluded that only three self-control scales have demonstrated evidence of reliability and validity in a variety of populations and with different types of behavioral outcomes: the Self-Control Scale [3], the Barratt Impulsiveness Scale [9], and the Low-Self- Control Scale [10]. Among them, one of the most widely used instruments that specifically focused on general self-control is the Brief Self-Control Scale (BSCS; [3]). This is a shorter 13-item unidimensional tool derived from the original 36 item Self-Control Scale (comprised of five factors assessing self-discipline, deliberate/non-impulsive action, healthy habits, work ethic, and reliability, developed from an extensive review on self-control processes and failures [11]). Since its publication, this brief measure has been used in more than 100 published studies with diverse and heterogeneous samples, and its utility has been demonstrated for assessing trait self-control and for predicting a variety of behavioral outcomes [12]. Additionally, several linguistic adaptations of the BSCS have been developed, including French [13], Chinese [14], Turkish [15], and German versions [16]. Nonetheless, an Italian version has yet to be developed and validated and hence, the present study aims to develop and test the the psychometric properties of an Italian version of the BSCS.

Some conflicting results have been reported about the factor structure of the BSCS. The measure was originally proposed to be unidimensional, but different factor solutions, which can be derived from the full scale or exclusion of some items, have been proposed. Employing the original English version of the BSCS, Ferrari et al. [17] found a two-factor structure for the 13 item that accounted for self-discipline and impulse control. De Ridder, et al. [18] proposed a 10-item version consisting of two factors, namely inhibition and initiation, and Maloney et al. [19] further suggests an eight item scale defined by the factors of restrain and impulsivity. The different linguistic adaptations also obtained different factor solutions. The French and German versions were deemed unidimensional [13, 12] while the Turkish version yielded two-factors, but partially different from the aforementioned two-factor models [15], and the Chinese version showed a good fit for a five-factor model [14]. In reference to these findings, two different approaches were proposed in the literature. On one hand, some authors suggested that none of the proposed alternatives seem to give better results in measuring trait self-control than the original unidimensional solution [12]. On the other hand, the difficulties encountered in replicating the BSCS factor structure led to the development of a BSCS version consisting of seven items allocated on two factors (namely, self-discipline and impulse control) [20]. In particular, the authors provided an extensive study of its psychometric properties attesting the replicability of the factor structure across different samples as well as demonstrating measurement invariance across demographic and clinical variables.

Starting from this premise, the internal structure of the Italian version was investigated using a confirmatory factor analysis method to test the original one-dimension structure, the two-factor structure proposed by Ferrari et al. [17], and the shortened two-factor models proposed by de Ridder et al. [18], Maloney at al. [19], and Morean et al. [20].

Once the factor structure of the scale has been tested and established, we aimed to test its invariance on the premise that a psychometrically sound measure should ensure that the construct is assessed similarly enough across groups of interest to allow making meaningful comparisons [21, 22]. Specifically, we tested the invariance of the Italian version of the BSCS across gender and age groups. Morean et al. [20] provided evidence of the measurement equivalence of their brief BSCS across gender, while partial invariance was reported by age, findings we expected to confirm. Moreover, since self-control can be a relevant risk-factor in some clinical populations (e.g., [23, 24]), the measurement equivalence of the scale should be examined in a clinical population. Specifically, chronic liver disease (CLD) patients were the sample of choice, given that self-control may play a relevant role both in the development of the disease and in the treatment adherence. Indeed, the leading causes of CLD may include disinhibited behaviors, such as excessive alcohol or food consumption, which induce cirrhosis and fatty liver disease [25]. Additionally, these individuals must follow medication adherence, laboratory testing and clinic visits, and a complex and variable regimen of dietary restrictions that require self-discipline [25]. Finally, the metrical equivalence of the Italian and the English version of the scale were investigated to determine if the scale, originally developed in English, maintains its latent factor structure once translated into Italian.

Along with validity based on the internal structure, a further aim was to provide evidence of the test-criterion validity of the Italian version of the BSCS. Specifically, we investigated the relationships of self-control with several psychological concepts and demographic variables seeking to replicate the nomological net observed for self-control, which can be detailed as follow.

Self-control has been related to various personality features (e.g., [3, 26]), but was mainly characterized by its relation with conscientiousness (i.e., Pearson’s *r* >.40 were reported) [27, 28], and mild to strong correlations were observed with individual dispositions, like mindfulness (i.e., tendency to be mindful in general daily life; [29]) and optimism. Indeed, self-control is linked to conscientiousness because individuals high in this trait have the tendency to set long-term goals, work in a disciplined way toward their goals, and prefer concrete- and rational-reasoned activities [30, 31]. Moreover, self-control has been associated to mindfulness because it predicts, to some extent, the maintenance of nonjudgmental and non-reactive states in the presence of desire [32], and to optimism, such that individuals who expect positive outcomes in the future may demonstrate willingness to exert self-control [33]. Furthermore, Morean et al. [20] reported the relationships with the Behavioral Inhibition and Activation Scales (BIS/BAS [34]) suggesting that self-control was not or only slightly correlated with the BIS/BAS scales with the exception of a negative moderate correlation with the BAS fun seeking scale [20]. Prior research studies suggest also that individuals with lower self-control would be more likely to be male and of younger age (e.g., [5, 35, 36]). Indeed, self-control seems to be less pronounced in adolescents and young men because they demonstrate greater sensation seeking, greater reward sensitivity, and lower punishment sensitivity when compared to their female or older counterpart [35, 36].

Finally, to extend the validity study, we explored the relationships with emotional intelligence (EI) that includes self-control, sociability, emotionality, and well-being [37]. We expected to find a strong positive relationship with the EI self-control, but also moderate positive correlations with sociability and emotionality, which both include self-regulation processes [3], and well-being, knowing that higher levels of self-control are linked to physical and psychological health (e.g., [5]).

## Methods

### Participants

#### Italian sample

A total of 1139 Italian speaking participants (age ranged from 18 to 76 years, *M* = 32.00 years, *SD* = 15.30, 57% females) were recruited for the study. Sampling was based on the “snowball” method [38], in which undergraduate students in a psychology course were invited to participate to the study and were also encouraged to recruit their acquaintances and relatives to participate. Participants provided informed consent, participation in the study was voluntary, and they did not receive compensation. The study was approved by the university’s local institutional review board (Commissione Etica per la Ricerca dell’Università degli Studi di Firenze, n. 31—prot. 127556).

#### English sample

The sample consists of 281 participants (ages ranged from 17 to 55 years; *M* = 18.46, *SD* = 2.72; 80% females). Undergraduate students, with English as their primary language, from a large Canadian university were invited to participate in the study. Upon signing up for the study, participants were directed to the online consent form and questionnaires. Participation in the study was voluntary and participants received a credit towards their psychology course. The study was approved by the university’s local institutional review board (Western University Non-Medical Research Ethics Board–NMREB, n. 111928).

#### Clinical sample

A sample of 217 Italian native outpatients referred to the liver disease clinic at a major Italian academic healthcare setting (ages ranged from 18 to 87 years; *M* = 60.12, *SD* = 14.86; 48% females) completed the study. Inclusion criteria included a diagnosis of chronic hepatitis B, chronic hepatitis C, alcoholic liver disease, hepatic steatosis, cirrhosis, liver cancer, autoimmune liver disease, primary sclerosing cholangitis, and/or primary biliary cholangitis. Additional demographic criteria included: (1) age ≥ 18 years, (2) Italian as the native language, (3) on active treatment, (4) no physical conditions that impair the patient's ability to complete the self-reported questionnaires and to release personal information through an interview by the staff, (5) absence of cognitive impairment, and (6) informed consent provided to the participation and to the analysis of deidentified data. Patients who did not meet the above inclusion criteria were excluded. Approval was obtained from the local ethics review board of the academic healthcare setting (Comitato Etico Locale Azienda Ospedaliero-Universitaria Careggi, n. 10574_oss).

Written informed consent was obtained from participants.

### Measures

The Brief Self-Control Scale (BSCS; [3]) consists of 13 items that yield a global assessment of dispositional self-regulatory behaviors using a 5-point scale (1 = not at all like me; 5 = very much like me). With the permission of the authors, the scale was translated into Italian by two Italian psychologists and the differences between their translations were discussed to address discrepancies and obtain a single version. This Italian version was back-translated into English by a native English-speaking person who was not familiar with the original version of the scale. The differences were discussed and addressed. The translated scale was then presented to three Italian university students to further check for readability and understandability. Specifically, they were asked to read the items to judge their clarity and indicate any unclear words or sentence meanings. The students reported the items were readable and comprehensible so that no further adjustments required. The final Italian version of the BSCS is reported in the S1 Appendix.

The Mindful Attention Awareness Scale (MAAS; [39, 40] for the 11-item Italian version) assesses experiences of acting automatically and without paying attention to the present moment (e.g. “I find it difficult to stay focused on what’s happening in the present”). Higher scores indicate higher mindfulness. Respondents rate how often they have this kind of experience on a 6-point Likert-type scale, ranging from “almost always” to “almost never”.

The Life Orientation Test—Revised (LOT-R; [41]; Italian version: [42]). The LOT-R measures dispositional optimism defined as a generalized expectancy of positive future outcomes. It consists of six items (e.g. “In uncertain times, I usually expect the best”) and four filler items answered on a 5-point Likert scale from “strongly disagree” to “strongly agree”. Higher scores indicate higher dispositional optimism.

The BIS/BAS ([35]; Italian version: [43]) assesses inhibition and activation, both of which motivate behavioral and emotional responses. The BIS (behavioral avoidance/inhibition) consists of seven items and measures sensitivity to aversive stimuli and avoidance of behaviours that might be associated with anxiety and fear, and that might produce punishment or frustration (e.g. “Criticism or scolding hurts me quite a bit”). The BAS (behavioral activation) consists of three different scales: the BAS Drive measures the motivation to follow one’s goals (four items, e.g. “I go out of my way to get things I want”), the BAS Reward Responsiveness assesses the sensitivity to pleasant reinforcements in the environment (five items, e.g. “When I'm doing well at something I love to keep at it”), and the BAS Fun Seeking measures the motivation to find novel and exciting rewards (four items, e.g. “I'm always willing to try something new if I think it will be fun”). Each item was rated on a four-point Likert scale from “not true at all for me” to “very true for me”. Higher scores indicate higher inhibition, motivation to follow one’s goals, sensitivity to pleasant reinforces, and motivation to find exciting rewards.

The Trait Emotional Intelligence Questionnaire-Short Form (TEIQue-SF; [44]; Italian version: [45]). The TEIQue-SF is a 30-item measure that evaluates global trait EI, though it can also be used to assess the four trait EI factors: Well-Being as a generalized sense of wellbeing, extending from past achievements to future expectation (six items, e.g. “On the whole, I’m pleased with my life”), Self-Control as emotion regulation, stress management, impulse control, adaptability, and self-motivation (six items, e.g. “I usually find it difficult to regulate my emotions”), Emotionality defined by emotion perception, trait empathy, and emotion expression (eight items, e.g. “Many times, I can’t figure out what emotion I'm feeling”), and Sociability as assertiveness, emotion management, and social awareness (six items, e.g. “I can deal effectively with people”). Participants responded to items using a seven-point Likert scale ranging from “completely disagree or strongly disagree” to “completely agree or strongly agree”. Higher scores indicate higher well-being, self-control, emotionality, and sociability.

The HEXACO-60 [46] consists of 60 items that measure six broad personality dimensions: Honesty-Humility (ten items, e.g. “I wouldn’t use flattery to get a raise or promotion at work, even if I thought it would succeed”), Emotionality (ten items, e.g. “I sometimes can’t help worrying about little things”), Extraversion (ten items, e.g. “In social situations, I’m usually the one who makes the first move”), Agreeableness (ten items, e.g. “I tend to be lenient in judging other people”), Conscientiousness (ten items, e.g. “I plan ahead and organize things, to avoid scrambling at the last minute”), and Openness to experiences (ten items, e.g. “People have often told me that I have a good imagination”). This set of six factors was recovered in independent standard lexical studies involving different languages, including Italian (see [47]). Each item rated on a five-point Likert scale from “strongly disagree” to “strongly agree”. Higher scores indicate higher honesty and humility, emotionality, extraversion, agreeableness, conscientiousness, and openness.

### Design and procedure

A descriptive observational research design was adopted. Specifically, it was a cross-sectional study with the exception of the test-retest for reliability testing (longitudinal repeated measures design).

The Italian version of the BSCS was presented to the Italian sample as part of an online questionnaire including other measures employed for validity testing. Specifically, participants were randomly asked to complete one of two different questionnaires developed to reduce time administration. One group (*n* = 639) were administered questionnaire that included the BSCS, the MAAS and the LOT-R. The remaining participants (*n* = 500) were given a questionnaire that included, along with the BSCS, the BIS/BAS, and the TEIQue-SF. Additionally, 227 participants completed the HEXACO-60. This subset was formed asking the participants of both groups to voluntary take part to an additional test administration and about 20% of them agreed to complete the HEXACO-60. Administration time ranged from 15 to 30 minutes. Similarly, the English sample completed the original English version of the scale on an online survey, while the clinical Italian sample received the paper-and-pencil version of the Italian BSCS. Administration time ranged from 5 to 10 minutes.

To investigate test-retest reliability, a small subset (*n* = 47) of the Italian undergraduate sample completed the BSCS again in a four- to five-week interval. This subset was formed asking the participants of the first group to voluntarily take part in this further administration and about 7% of these participants agreed to complete the BSCS after one month from the first administration. The smaller sample size can be considered adequate for the reliability study, given that a high value (ρ < 0.8) with a small width of the relative CI (*w* = 0.2) was expected [48]

### Analysis strategy

Prior to conducting the analyses, we examined the missing values in the data. For each item, we then computed the percentage of missing responses to ensure that the missing data did not exceed 10% of the total answers. Listwise deletion was used for these cases. Otherwise, the arithmetic mean of each item was used to replace the missing data [49].

**Fig 1:**
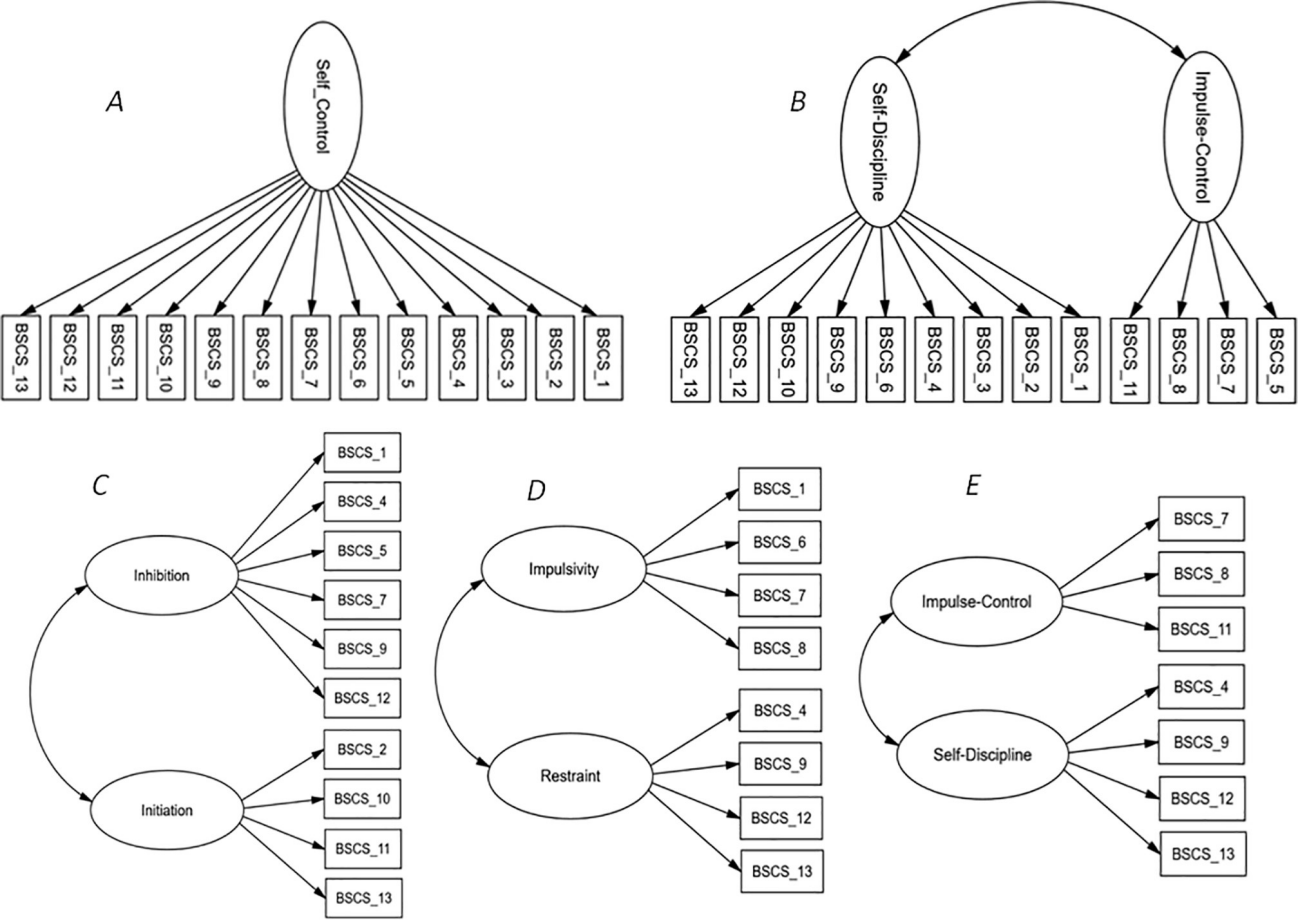
Brief Self-Control Scale (BSCS) factor models proposed by *A*: Tangney et al.; *B*: Ferrari et al.; *C*: De Ridder et al.; *D*: Maloney et al.; *E*: Morean et al.

To assess the factor structure of the BSCS, confirmatory factor analysis (CFA) was conducted testing five different models (Fig 1): the original unidimensional model [3], the two-factor model, which includes all the BSCS items [17], and the shortened two-factor models proposed by de Ridder et al. [18] Maloney at al. [19], and Morean et al. [20], respectively. Item 4 was used as the marker variable for model identification because it was included in all the different tested models. The Comparative Fit Index (CFI), Tucker-Lewis index (TLI), Standardized Root Mean Square Residual (SRMR), and Root Mean Square Error of Approximation (RMSEA) were used to evaluate the goodness-of-fit. Specifically, SRMR and RMSEA values lower than .08 would suggest an adequate model fit, and CFI and TLI values in the range of .90 and .95 would suggest moderate to excellent model fit [49, 50]. Finally, Akaike Information Criterion (AIC) was used to compare different models, with lower AIC values indicating better fit.

Multi-group CFA [51, 52, 21] was also used to evaluate whether the factor structure was consistent across gender, age, clinical status, and language. Specifically, factorial invariance exists when: a. the construct is associated with the same set of items in each group (i.e., configural invariance), b. the relationships between the construct and the items, as represented by factor loadings, is not significantly different across group variables (i.e., metric invariance), c. both the factor pattern coefficients and the intercepts are equal across groups (i.e., scalar invariance), d. the error terms do not differ across groups (i.e., strict invariance). Strict factorial invariance is not necessary to make mean comparisons among groups but the assessment of scalar invariance is needed, given that mean differences in scores reflect true differences in the measured construct due to group membership [21, 22]. Otherwise differences might be attributable to measurement biases in specific items. Nonetheless, partial metric and partial scalar invariance can be established by allowing a subset of the factor loadings and intercepts to vary freely across groups, while constraining the other ones to equality [53, 21]. Although establishing full scalar invariance is preferable, partial scalar invariance also allows for mean differences to be compared meaningfully (e.g., [21]).

To assess measurement invariance in the factor structure of the BSCS, preliminary single-group CFAs were conducted to examine separately the factorial structure of the BSCS in each group [54, 55]. Specifically, the samples were defined as follows. Splitting by gender, produced a female sample (reference group) consisting of 647 cases (age: *M* = 32.08, *SD* = 15.45) and a male sample of 492 cases (age: *M* = 32.11 years, *SD* = 15.04). For age, the sample was split based on the median value (22 years), excluding the cases equal to the median (*n* = 86) and then we obtained a younger adult sample that we used as the reference group (*M* = 20.33 years, *SD* = 0.78, 68% female, *n* = 492) and an older adult sample (*M* = 44.00 years, *SD* = 13.96, 49% female, *n* = 561). To obtain comparable samples for invariance across clinical status, we randomly selected a subset of adults over 40 years of age (*M* = 53.58, *SD* = 5.48, 60% female, *n* = 364), which was used as the reference group. Similarly, for language invariance, we randomly selected a sample of young Italian university students (*M* = 19.75, *SD* = 0.61, 69% female, *n* = 265) that was as similar as possible in size, age and gender to the English sample used as the reference group (*M* = 18.16, *SD* = 0.93, 80% female, *n* = 274). Sample random selection was made using the SPSS–Version 26. Taking into account the number of items (i.e., seven or thirteen) and dimensions (i.e., one or two), all the aforementioned sample sizes were adequate to perform both single and multi-group analyses [56].

Subsequently, a hierarchically nested series of CFAs were applied [52]. An unconstrained model, labelled Model 0, was used to test configural invariance. Then, three more restrictive models were tested, which include: Model 1 in which factor loadings were constrained to be equal across groups to test metric invariance, Model 2 in which factor loadings and intercepts were constrained to be equal across groups to test scalar invariance, Model 3 in which factor loadings, intercepts and error terms were constrained to be equal across groups to test strict invariance. Any subsequent restriction was applied only if the previous restriction was allowed, and the comparison was done between the last fitting model and the following more restricted one [57, 58]. Models were compared using the chi-square-based likelihood ratio difference (Δχ^2^), the Comparative Fit Index difference (ΔCFI), and Root Mean Square Error of Approximation difference (ΔRMSEA). A significant Δχ^2^ value along with a ΔCFI value ≤.01 [50, 59] and a change ≤.015 in RMSEA would indicate invariance [57].

Preliminary analysis of the data showed that the multivariate Mardia’s coefficients ranged from 16.2 to 27.6 across samples. These values were lower than the criterion value ≤ 30 [60], indicating normality of responses to the BSCS items. Thus, all one-group and multi-group CFAs were conducted applying Maximum likelihood estimation using the software program *R* (version 6.12) and its package *Lavaan* [61].

Reliability was tested as internal consistency and temporal stability. McDonald’s Omega coefficient was used for the internal consistency. Omega has been shown to be a more sensitive index of internal consistency compared to Cronbach’s alpha under violations of tau-equivalence (e.g., [62]), or when some item error terms are correlated [63]. McDonald’s ω ≥ .70 is considered satisfactory. Temporal stability (i.e., test-retest) was tested computing intra-class correlations.

Validity was tested using bivariate correlations relating the BSCS scale score with personality and related psychological concept measures. Additionally, Bayesian independent sample *t*-tests were used to evaluate the extent to which group membership (in all subgroups for which scalar [or partial scalar] measurement invariance was established) was associated with differences in self-control. The Bayesian approach can quantify relative evidence for both H1 and H0, and the magnitude of this evidence is presented as an easy-to-interpret odds ratio. Since we hypothesized that individuals with poor self-control would be more likely to be male and young, we tested the following one-sided alternative hypotheses: male < female and young < adults. Specifically, a BF value between 1 and 3 is considered weak evidence for the alternative hypothesis, a BF between 3 and 10 is considered moderate evidence, and a BF greater than 10 is considered strong evidence (e.g., [64, 65]). In addition, the 95% credible interval of the effect size δ was computed. All the Bayesian tests were performed with JASP 0.10 [66].

## Results

Minimal data were missing across all variables. For each item of the BSCS, the missing values remained under 5% of the total cases in the sample, and no case had more than two missing responses out of thirteen; thus, the arithmetic mean of each item was used to replace the missing data.

### Internal structure

Model fit statistics based on the CFA performed on the different BSCS proposed models are reported in Table 1. Results suggested that all the tested models had an unsatisfactory fit with the exception of the Morean et al.’s two-factor model [20] once—as suggested by the modification index (MI = 57.45)—a covariance was considered between item 9 (“Pleasure and fun sometimes keep me from getting work done”) and item 11 (“I am able to work effectively toward long-term goals”). Consistently, the lower AIC value was observed for this model, which suggested this model was optimal compared to other tested models for the Italian version of the scale. Item-loadings were all significant at *p* < .001. The first factor loadings ranged from .47 to .72 (self-discipline: .54, .52, .72, and .47) and the second factor loadings ranged from .33 to .65 in (impulse control: .62, .65, and .33). The correlation between the two factors self-discipline and impulse control was .59. Then, the following invariance analyses of the Italian version of the BSCS (BSCS-IT) were based on this model.

**Table 1:** Fit statistics of the alternative BSCS models in the Italian sample.

*Model*	*χ*^*2*^*(df)*	*CFI*	*TLI*	*RMSEA (90% CI)*	*SRMR*	*AIC*
One-factor	723.86 (65)	.780	.737	.094 [.088, .101]	.065	775.88
Two-factors						
Ferrari et al. [17]	596.98 (64)	.822	.784	.086 [.079, .092]	.058	650.98
de Ridder et al. [18]	411.39 (34)	.818	.759	.099 [.081, .107]	.063	453.39
Maloney et al. [19]	154.47 (19)	.917	.878	.079 [.068, .091]	.041	188.47
Morean et al. [20]	116.66 (13)	.909	.853	.084 [.070, .098]	.047	146.66
Morean et al. *	58.082 (12)	.960	.929	.058 [.044, .073]	.030	90.03

*The model included a covariation between item 9 and 11; *N* = 1139; *df* = degrees of freedom; CFI = comparative fit index; TLI = Tucker-Lewis index, RMSEA = root mean square error of approximation; 90%CI = 90% confidence interval around RMSEA; SRMR = standardized root mean square residual. AIC = Akaike Information Criterion.

### Measurement invariance

First, Morean et al.’s modified model (i.e., including the covariation between item 9 and 11) was tested in each group used for invariance testing (Table 2). Results confirmed that the model holds in all the subsamples derived from the Italian sample (i.e., males, female, young adults, adults, Italian university students, and non-clinical older adults), and we showed that the model had a good fit in the English sample and the clinical sample. Specifically, all the different BSCS models were tested in the clinical sample. Results (see S1 Table) confirmed that the Morean et al.’s two-factor model was the best fitting model.

**Table 2:** Fit statistics of the brief Morean at al.’s BSCS model in each sample used for invariance testing.

*Sample*	*χ*^*2*^*(df)*	*CFI*	*TLI*	*RMSEA [90% CI]*	*SRMR*
Male (*n* = 492)	27.76 (12)	.961	.931	.052 [.026, .077]	.037
Female (*n* = 647)	43.39 (12)	.960	.930	.063 [.043, .084]	.033
Young (*n* = 492)	22.27 (12)	.983	.970	.042 [.011, .068]	.027
Adult (*n* = 561)	44.11 (12)	.937	.890	.069 [.048, .092]	.038
Italian University (*n* = 265)	18.07 (12)	.978	.962	.044 [.000, .083]	.034
English University (*n* = 274)	31.26 (12)	.959	.928	.077 [.044, .110]	.038
Non Clinical (*n* = 364)	19.58 (12)	.978	.962	.042 [.000, .074]	.031
Clinical (*n* = 217)	27.64 (12)	.944	.902	.078 [.039, .116]	.048

All the analyses included a covariation between item 9 and 11. *df* = degrees of freedom; CFI = comparative fit index; TLI = Tucker-Lewis index, RMSEA = root mean square error of approximation; 90%CI = 90% confidence interval around RMSEA; SRMR. = standardized root mean square residual.

The overall and comparative fit statistics of invariance models are presented in Table 3.

**Table 3:** Fit statistics of the Brief BSCS invariant models across genders, ages, clinical status, and languages.

*Model*: *Invariance level*	*χ*^*2*^*(df)*	*CFI*	*RMSEA*	*Model Comparison*	*Δχ*^*2*^	*Δdf*	*p*	ΔCFI*	ΔRMSEA*
*Gender*									
Model 0: Configural (unconstrained)	70.14 (24)	.960	.041	-	-	-	-	-	-
Model 1: Metric (measurement weights)	82.96 (29)	.953	.040	Model 1 –Model 0	12.82	5	.025	.007	.001
Model 2: Scalar (measurement intercepts)	124.19 (36)	.924	.046	Model 2 –Model 1	41.22	7	< .001	.029	.006
Model 2a: Partial Scalar (Item 4 τ free)	101.86 (35)	.943	.041	Model 2a –Model 1	18.90	6	.004	.010	.001
Model 3: Strict (measurement error)	122.16 (42)	.931	.041	Model 3 –Model 2a	20.30	7	.004	.012	.000
*Age*									
Model 0: Configural (unconstrained)	66.38 (24)	.962	.041	-	-	-	-	-	-
Model 1: Metric (measurement weights)	80.92 (29)	.953	.041	Model 1 –Model 0	14.54	5	.013	.009	.000
Model 2: Scalar (measurement intercepts)	178.26 (36)	.872	.061	Model 2 –Model 1	97.34	7	< .001	.081	.020
Model 2a: Partial Scalar (Item 9 τ free)	113.24 (35)	.930	.046	Model 2a –Model 1	32.32	6	< .001	.023	.005
Model 2b: Partial Scalar (Item 9 and Item 11 τs free)	96.42 (34)	.944	.042	Model 2b –Model 1	15.50	5	.008	.009	.001
Model 3: Strict (measurement error)	114.80 (39)	.932	.043	Model 3 –Model 2b	18.38	5	< .001	.012	.001
*Clinical status*									
Model 0: Configural (unconstrained)	47.41 (24)	.963	.058	-	-	-	-	-	-
Model 1: Metric (measurement weights)	50.61 (29)	.966	.051	Model 1 –Model 0	3.20	5	.670	.003	.007
Model 2: Scalar (measurement intercepts)	91.28 (34)	.909	.076	Model 2 –Model 1	40.67	5	< .001	.057	.026
Model 2a: Partial Scalar (τ Item 11 free)	58.91 (33)	.959	.052	Model 2a –Model 1	8.31	4	.080	.007	.001
Model 3: Strict (measurement error)	87.98 (40)	.924	.064	Model 3 –Model 2a	29.06	7	< .001	.035	.012
*Language*									
Model 0: Configural (unconstrained)	49.33 (26)	.966	.044	-	-	-	-	-	-
Model 1: Metric (measurement weights)	58.51 (29)	.961	.044	Model 1 –Model 0	9.18	5	.102	.005	.000
Model 2: Scalar (measurement intercepts)	180.85 (36)	.807	.087	Model 2 –Model 1	131.52	7	< .001	.154	.043

*Differences are reported in absolute value; *df* = degrees of freedom; CFI = comparative fit index; RMSEA = root mean square error of approximation; 90% CI = 90% confidence interval around RMSEA; Δχ^2^ = difference in χ^2^; Δ*df* = difference in degrees of freedom; ΔCFI = Difference between CFIs; ΔRMSEA = difference in root mean square error of approximation.

Multi-group CFAs conducted to test the measurement equivalence of the BSCS-IT across genders and ages showed an adequate fit of Model 0 and Model 1. When comparing these models, CFI and RMSEA values were lower than .01 and .015 respectively, indicating metric invariance across genders and ages. Scalar invariance was not demonstrated for gender. Nonetheless, as suggested by the modification index (MI = 9.79), if group-specific intercepts of item 4 were estimated, the decrement in fit between scalar and metric invariance models was of ΔCFI = .010, accompanied by a change in RMSEA = .001, demonstrating partial scalar invariance. Comparing this model with Model 3, strict invariance was not demonstrated (i.e., ΔCFI = .012).

Similarly, if the intercepts of item 9 and item 11 were unconstrained (MI = 47.34 and MI = 11.75, respectively), and freely estimated for the young and adult groups, the CFI difference was .009 accompanied by a change in RMSEA of .001, suggesting age partial scalar invariance. Comparing this model with Model 3, strict invariance was not demonstrated (i.e., ΔCFI = .012).

The multi-group CFA was conducted to test the measurement equivalence of the BSCS-IT across clinical status. Results showed an adequate fit of Model 0 and Model 1. Δχ^2^ was not significant when comparing these models and the changes in CFI and RMSEA values were .003 and .007, respectively, indicating metric invariance. Scalar invariance was not demonstrated. Then, as suggested by the modification index (MI = 22.53), the intercepts of item 11 were unconstrained. The decrement in model fit between scalar and metric invariance models was ΔCFI = .007 and ΔRMSEA = .001, demonstrating partial scalar invariance. Comparing this model with Model 3, strict invariance was not demonstrated (i.e., ΔCFI = .035).

Finally, a multi-group CFA was conducted to test the measurement equivalence of BSCS-IT across the English and Italian versions. Results showed an adequate fit of Model 0 and Model 1. Δχ^2^ was not significant and the difference in CFI and RMSEA values was .005 and < .001, respectively, indicating metric invariance. Scalar invariance and partial scalar invariance were not demonstrated (i.e., more than three item intercepts have to be unconstrained to reach a non-significant decrement in the model fit).

### Reliability

For internal consistency, McDonald’s ω for the BSCS-IT was .70. No increases in omega values were observed if any of the individual items were removed from the scale. McDonald’s ω for the impulse control and self-discipline factors were .66 and .58, respectively. Although these values fall below the acceptable cut-offs for reliability indices, no increases in omega values were observed if any of the individual items were removed from the factor. Mean inter-item correlations were .32 and .27, respectively, which can be considered adequate [67]. Test–retest reliability was good in a small subset of individuals four to five weeks after initial assessment. The average measure ICC was .92 [95% CI: .86-.96] for the total score, .91 [95% CI: .85-.95] for the impulse control factor, and .84 [95% CI: .71-.91] for the self-discipline factor.

### Validity

For validity analysis, one case was excluded in the first subsample that completed the MAAS and LOT-R scales and two cases in the second subsample that completed the BIS/BAS and TEIQue-SF scales because more than 10% of data were missing for responses in these additional scales. Thus, analyses were conducted on *N* = 638 and *N* = 498 cases, respectively.

Means, standard deviations, and bivariate correlations of the BSCS-IT with subscales of the HEXACO-60 and related psychological concepts were computed (Table 4). A significant moderate positive correlation was found with conscientiousness and mindfulness. Extraversion and optimism were also significantly and positively correlated, and the effect size of the correlations was moderate. The association with emotional intelligence (Table 5) suggested that all the four factors were correlated (*p* < .001) with self-control. Specifically, the higher correlation was observed for the self-control dimension (.40), but also emotionality, sociability and well-being were positively and moderately correlated with the BSCS-IT. As for the BIS/BAS, only the BAS Fun Seeking dimension was correlated with the BSCS-IT (*p* < .001). The correlation was negative and moderate in size.

**Table 4:** Means, standard deviations, internal consistency coefficients, and bivariate correlates between the BSCS-IT, HEXACO, MASS, and LOT-R.

	*M (SD)*	*Range*	1.	2.	3.	4.	5.	6.	7.	8.	9.	10.	11.
1. BSCS-IT	23.96 (4.80)	4–20	.*71*										
2. BSCS-IT Impulse Control	14.32 (3.30)	3–15	.88**	.*67*									
3. BSCS-IT Self-discipline	9.65 (2.44)	9–35	.77**	.39**	.*62*								
4. Honesty-Humility	36.60 (6.60)	14–50	.14*	.15*	.09	.*75*							
5. Emotionality	34.80 (5.90)	17–49	-.11	-.12	-.06	.08	.*78*						
6. Extraversion	30.70 (7.80)	12–48	.20**	.12	.25**	.01	-.26**	.*83*					
7. Agreeableness	31.10 (6.40)	10–49	.12	.15*	.04	.15*	-.11	.04	.*77*				
8. Conscientiousness	37.80 (6.10)	20–50	.45**	.41**	.39**	.11	.04	.13*	.09	.*76*			
9. Openness to experience	34.80 (6.30)	18–49	.01	.01	.03	.12	-.08	.12	-.01	-.02	.*71*		
10. MAAS	27.70 (9.74)	11–62	.35**	.40**	.15*	.17*	-.15*	.16*	.09	.17*	-.10	.*80*	.
11. LOT-R	19.10 (4.75)	6–30	.22**	.19*	.19*	.04	-.33**	.57**	.12	.17	.08	.24**	.*85*

*N* = 227 for the HEXACO and *N* = 638 for MAAS and LOT-R. McDonald’s ω (in *italics*) are in diagonal.

* *p* < .01 and

** *p* < .001 (adjusted level of significance for Type 1 error). BSCS-IT = Brief Self-Control Scale- Italian version; MAAS = Mindful Attention Awareness Scale; LOT-R = Life Orientation Test Revised.

**Table 5:** Means, standard deviations, internal consistency coefficients, and bivariate correlates between the BSCS-IT, BIS/BAS, and TEIQue-SF.

	*M (SD)*	*Range*	1.	2.	3.	4.	5.	6.	7.	8.	9.	10.	11.	12.
1. BSCS-IT	23.50 (4.69)	4–20	.*69*											
2. BSCS-IT Impulse Control	14.00 (3.31)	3–15	.90**	.*63*										
3. BSCS-IT Self-discipline	9.50 (2.22)	7–35	.77**	.41**	.*53*									
4. BIS	20.78 (3.91)	9–28	-.06	-.06	-.04	.*76*								
5. BAS Reward	16.78 (2.28)	10–20	.03	-.02	-.09	.12*	.*64*							
6. BAS Fun Seeking	10.59 (2.51)	5–16	-.45**	-.50**	-.20**	-.12**	-.28**	.*65*						
7. BAS Drive	10.69 (2.42)	4–16	-.02	-,07	.08	.20**	.37**	.43**	.*70*					
8. TEIQue-SF Emotionality	39.87 (7.46)	16–56	.30**	.26**	.26**	-.02	.16**	-.03	.14*	.*66*				
9. TEIQue-SF Self-Control	26.14 (5.95)	6–42	.40**	.39**	.28**	-.46**	.02	-.15**	.12*	.28**	.*65*			
10. TEIQue-SF Well-Being	30.44 (6.45)	6–42	.27**	.23**	.24**	-.31**	.27**	.12*	.25**	.42**	.44**	.*81*		
11. TEIQue-SF Sociability	26.89 (6.10)	10–42	.24**	.20**	.20**	-.29**	.24**	.16**	.39**	.39**	.33**	.41**	.*68*	
12. TEIQue-SF	142.66 (22.06)	71–196	.42**	.37**	.35**	-.35**	.24**	.05	.34**	.73**	.67**	.79**	.70**	.*87*

*N* = 498. McDonald’s ω (in *italics*) are in diagonal.

* *p* < .01 and

** *p* < .001 (adjusted level of significance for Type 1 error). BSCS-IT = Brief Self-Control Scale–Italian version; BIS = Behavioral Inhibition Scale; BAS = Behavioral Activation Scale; TEIQue-SF = Trait Emotional Intelligence Questionnaire-Short Form.

An inspection of the two factors, by and large suggested the same pattern of correlations observed for the total score with few exceptions. A moderate positive correlation was found with extraversion and the self-discipline dimension (*p* < .001), while the impulse control was not significantly correlated with this personality trait. A weak positive correlation was also found between agreeableness and impulse control. Finally, only the BAS Fun Seeking dimension was large and negatively correlated with the impulse control dimension while the correlation with self-discipline was moderate.

For known-group validity, the one-sided *t*-test showed that the resulting BF value was 7.70 for gender, indicating moderate evidence in favor of alternative hypothesis (M_male_ < M_female_). The 95% credible interval for δ ranged from -0.29 to -0.06, indicating a 95% probability that the effect in the population is in this interval. Specifically, one-sided Bayesian *t*-test showed that the BF value was 32.83 for impulse control, indicating strong evidence in favor of alternative hypothesis (M_male_ < M_female_), and the 95% credible interval for δ ranged from -0.31 to -0.08. The BF value was 0.21 for self-discipline, indicating there was no difference between groups [95% CI: -.18-.00].

The BF value of 168.01 observed for age indicated strong evidence in favor of alternative hypothesis (M_young_ < M_adult_) and the 95% credible interval for δ ranged from -0.34 to -0.11. Specifically, one-sided Bayesian *t*-test showed that the BF value was 2887.24 for impulse control, indicating strong evidence in favor of alternative hypothesis (M_young_ < M_adult_). The 95% credible interval for δ ranged from -0.38 to -0.15, which means that there is a 95% probability that the effect in the population is in this interval. The BF value was 0.31 for self-discipline indicating there was no difference between groups [95% CI: -.20-.01].

## Discussion

The current study examined the psychometric properties of an Italian adaptation of the Brief Self-Control Scale (Tangney et al. [3]). We assessed the factor structure of the scale in a large sample of Italian-speaking participants. Since divergent results have been reported with regards to the BSCS factor structure, different factor solutions were tested in the present study. Results suggested that the best fitting model was proposed by Morean et al, [20], consisting of seven items allocated on two highly correlated factors (self-discipline and impulse control), which allow for a global assessment of dispositional self-regulatory behaviors. For the Italian version, the covariation between item 9 and 11 were likely a result of their proximity and the similar wording. Specifically, in the Italian translation the word “termine” [term]” was used in both items and this may have contributed to excess of covariation between them. This aspect along with the invariance results (see below) might suggest eliminating one or both these items but unfortunately, further shortening of the measure demonstrated lower reliability and validity when compared to the 7-item version. For this reason, we opted to include the covariation instead of item elimination.

The gender, age, clinical status, and language measurement invariance of the BSCS-IT were tested to ensure that the construct is assessed similarly enough across these groups. Results showed that the BSCS-IT can be employed with adult respondents of different ages and both genders to make unbiased comparisons. Indeed, we confirmed the gender and age metric invariance of the scale (i.e., the construct structure and the relationships between the construct and the items are equal across groups) and observed a partial scalar invariance that allows for mean differences to be compared meaningfully (e.g., [21]). In particular, we identified the same biased items reported by Morean et al., [20] for age, namely item 9 and item 11 (“Pleasure and fun sometimes keep me from getting work done” and “I am able to work effectively toward long-term goals”), confirming that these two items are differently interpreted by younger and older respondents. Specifically, we might suggest that the term “work” has a different meaning for young people, which could mainly refer to studying, while middle-aged adults may think of career-related endeavors. As such, self-control might have a different impact for young adults compared to middle-aged adults if pleasure and fun restrict one from focusing on studies and work, respectively. Similarly, we can presume that long-term goals might have a different meaning for younger and middle-aged adults for several reasons, including that middle-aged adults may have reached their long term objectives or have a different perspective in terms of achievement, while long-term objectives might be not well defined or too far into the future to be reached for young people (e.g., to find a job, get married). Finally, we identified also item 4 (“I do certain things that are bad for me, if they are fun”) as non-invariant across male and female respondents. A tentative explanation is that cultural gender patterns might influence the answer to this specific item. Indeed, women may be judged more harshly than men for adopting unhealthy, unconventional, and risky behaviors for fun [68].

Given that self-control can be a relevant risk-factor in some clinical populations, we aimed to test the measurement equivalence of the Italian version of the BSCS in a clinical sample to assure that the scale maintains its characteristics for clinical respondents and, as such, it can be used in assessment protocols before and during the treatment to measure and monitor patients’ self-control. Specifically comparing non-clinical participants with chronic liver disease patients, we found the construct structure and the relationships between the construct and the items are equal across groups (i.e., metric invariance). Moreover, partial scalar invariance was demonstrated. Partial invariance suggests that item 11 works differently in the two groups. Having in mind that this item refers to working on long-term goals, the older age of many patients and the impact of the disease may be relevant and provide explanations for these results.

Finally, since the scale was developed in English, it is important to ensure that the scale maintains its latent factor structure across languages. Although scalar invariance was not demonstrated, and therefore direct comparison between groups cannot be done, we provided evidence that English-speaking and Italian-speaking individuals use the same conceptual framework to answer the items of the scale. Specifically, the invariance testing provided evidence of the conceptual equivalence of the underlying latent variable across groups and the use of identical indicators to measure this latent variable, namely self-control as defined by impulse control and self-discipline.

Reliability was tested as internal consistency and temporal (re-test) stability, and both suggested an adequate reliability of the total BSCS-IT. However, each single factor showed weaker internal consistency, which could be a result of the small number of items per factor. Nonetheless, their temporal stability was good.

Validity evidence was provided by examining the relationships between the Italian version of the BSCS and other measures of related psychological concepts and demographic variables, including personality trait, individual dispositions, age and gender. By and large, results offer support for its nomological net. In line with published research [27, 28, 3], self-control, as measured by the BSCS-IT, showed the stronger correlations with conscientiousness when compared to the other major personality dispositions that were uncorrelated or weakly correlated with self-control. As expected, a positive and small in size correlation confirmed that optimism and self-control are distinct traits that have important self-regulatory functions [69] and they are partially related. Specifically, individuals exert self-control because there is expectancy and valence in the positive outcomes that one desires [33]. Similarly, a positive moderate correlation confirmed that self-control was related to dispositional mindfulness, which predicts resistance to act on impulses driven by desire [32]. In line with Morean et al. [20], we observed a nonsignificant correlation with Reward Responsiveness and a negative moderate correlation with the BAS Fun Seeking scale. The direction and the effect size of this relationship was not surprising because this scale conceptualizes the motivation to find novel exciting rewards similarly to the BSCS measures the resistance to behaviours associated with fun, pleasure, and immediate rewards. Partially different results were obtained for the BIS and BAS Drive scales because this study did not obtain the modest correlations (negative for BIS and positive for BAS Drive) reported by Morean et al. [20]. However, the current results are not unexpected, given that the BIS and BAS Drive scales assess the avoidance of behaviours that might be associated with anxiety or fear and motivational aspects, which are not included in the BSCS. In line with prior research (e.g., [5, 35, 36]), poorer self-control was found in males and younger individuals, compared to their female and older counterparts, respectively. As previously reported [20], these differences may be attributable to differences in impulse control. Young men are more likely to be greater sensation seekers, more sensitive to immediate and exciting rewards, and less sensitive to negative consequences when compared to their female counterpart [35, 36]. The differences found in impulse control may be explained taking into account these characteristics.

Finally, the Italian version of the BSCS showed a positive correlation with EI self-control, which assesses emotion regulation, stress management, impulse control, adaptability, and self-motivation. The correlation was not as large in size as it might be expected, most likely because there is only a partial overlap between the two concepts (e.g., stress management and self- motivation are not included in the BSCS). Additionally, we found positive and small correlations with sociability and emotionality dimensions, which refer to self-regulation processes and emotion management, and well-being. This result was expected because higher levels of self-control are usually linked to better physical and psychological health [5].

While this study provides some evidence that the shortened BSCS can be used as a measure of self-control in the Italian context as well as providing broader support for the robustness of the measure, there are some limitations to the present effort and, consequently, suggestions for future research. First of all, whereas metric invariance across age, gender, clinical status, and language was demonstrated, scalar invariance was only partially achieved, indicating that some items are differentially understood depending on group membership and, as a consequence, items 4, 9 and 11 should not be considered when making comparisons. Taken together, these findings suggest the need for rewording or partially modifying these items in future investigations. Secondly, reliability was adequate, but it would be preferable to have stronger internal consistency and perhaps the aforementioned item changes may help in improving both construct validity based on the internal structure and internal consistency. Third, we tested the invariance across clinical status on a specific clinical sample (i.e., patients with chronic liver disease) but further studies are needed to generalize the possibility to use the scale in patients affected by other pathologies in which self-control should be measured and monitored (e.g., obesity and diabetes). Finally, two administration formats (pencil-and-paper and online surveys) were used; the subsample used to test temporal stability was small in size and exclusively made up of undergraduate students. Canadian students were rewarded for their participation with a credit towards their psychology course while Italian students were not. All these methodological dissimilarities should be avoided in future investigations to confirm and strengthen the current results.

Overall, this study provides preliminary evidence that the BSCS-IT has adequate psychometric properties. As such, the measure could be used in research focused on self-control and potentially offers added value in measuring self-control for clinical purposes.

## Supporting information

S1 Table (DOCX)

S1 Appendix (DOCX)

S1 Data (ZIP)
