# Supplementary material for: Measuring self-control across gender, age, language, and clinical status: A validation study of the Italian version of the Brief Self- Control Scale (BSCS)
Source: PLoS One. 2020 Aug 21;15(8):e0237729. doi: 10.1371/journal.pone.0237729 (PMC7446922; doi:10.1371/journal.pone.0237729)
Supplement: S1 Table — (DOCX) [file pone.0237729.s001.docx]

Table 1a. *Fit statistics of the alternative BSCS models in the clinical sample.*

| *Model* | *χ^2^(df)* | *CFI* | *TLI* | *RMSEA (90% CI)* | *SRMR* | *AIC* |
| --- | --- | --- | --- | --- | --- | --- |
| One-factor | 243.00 (65) | .741 | .690 | .113 [.098, .128] | .084 | 295.00 |
| Two-factors |  |  |  |  |  |  |
| Ferrari et al. | 164.89 (64) | .853 | .821 | .085 [.069, .102] | .070 | 218.89 |
| de Ridder et al. | 96.94 (34) | .846 | .797 | .093 [.071, .114] | .065 | 138.94 |
| Maloney et al. | 108.04 (19) | .795 | .697 | .147 [.121, .175] | .083 | 142.04 |
| Morean al. | 29.39 (13) | .941 | .905 | .076 [.039, .113] | .052 | 59.39 |

*Note*: *N* = 217; *df* = degrees of freedom; CFI= comparative fit index; TLI: Tucker-Lewis index, RMSEA = root mean square error of approximation; 90%CI = 90% confidence interval around RMSEA; SRMR: standardized root mean square residual. AIC = Akaike Information Criterion.
