# Supplementary material for: Measuring self-control across gender, age, language, and clinical status: A validation study of the Italian version of the Brief Self- Control Scale (BSCS)
Source: PLoS One. 2020 Aug 21;15(8):e0237729. doi: 10.1371/journal.pone.0237729 (PMC7446922; doi:10.1371/journal.pone.0237729)
Supplement: S1 Appendix — (DOCX) [file pone.0237729.s002.docx]

**Appendix**

Italian Version of the Brief Self-Control Scale (BSCS-IT)

| *Item* | Original Version | Italian Version |
| --- | --- | --- |
| *1* | I have a hard time breaking bad habits (R) | Ho difficoltà a interrompere le cattive abitudini |
| *2* | I am lazy (R) | Sono pigro/a |
| *3* | I say inappropriate things (R) | Dico cose inappropriate |
| *4* | **I do certain things that are bad for me, if they are fun** (R) | **Faccio alcune cose che mi fanno male, se sono divertenti** |
| *5* | I refuse things that are bad for me | Rifiuto cose che mi fanno male |
| *6* | I wish I had more self-discipline (R) | Vorrei avere una maggiore auto-disciplina |
| *7* | **I am good at resisting temptation** | **Sono brava/o a resistere alla tentazione** |
| *8* | **People would say that I have iron self-discipline** | **La gente direbbe che ho una ferrea auto-disciplina** |
| *9* | **Pleasure and fun sometimes keep me from getting work done** (R) | **Svago e divertimento a volte mi impediscono di portare a termine il lavoro** |
| *10* | I have trouble concentrating (R) | Ho difficoltà a concentrarmi |
| *11* | **I am able to work effectively toward long-term goals** | **Sono in grado di lavorare efficacemente verso obiettivi a lungo termine** |
| *12* | **Sometimes I can’t stop myself from doing something, even though I know it is wrong** (R) | **A volte non riesco a impedirmi di fare qualcosa, anche se so che è sbagliato** |
| *13* | **I often act without thinking through all the alternatives** (R) | **Spesso agisco senza pensare a tutte le alternative** |

*Note*: in bold the items of the short BSCS proposed by Morean et al. (2014).
